# Anesthetic management of a gross type C tracheoesophageal fistula by balloon catheter via tracheostoma in an infant with trisomy 18: A Case Report

**DOI:** 10.1097/MD.0000000000048614

**Published:** 2026-05-08

**Authors:** Masato Ryo, Yusuke Ishida, Maiko Hosokawa, Kiyoko Bito, Katsunori Oe

**Affiliations:** a Department of Anesthesiology, St. Luke’s International Hospital, Tokyo, Japan; b Department of Anesthesiology, Showa Medical University School of Medicine, Shinagawa-ku, Tokyo, Japan.

**Keywords:** airway management, esophageal atresia, tracheoesophageal fistula, tracheostomy, trisomy 18

## Abstract

**Rationale:**

Gross Type C congenital esophageal atresia is associated with tracheoesophageal fistula (TEF). Perioperative airway management, including prevention of aspiration of gastric contents, is important. Generally, the TEF is blocked by 1-lung intubation of the dependent lung, or directly by insertion of a balloon catheter. This case report describes successful management of TEF by insertion of a balloon catheter via tracheostoma in an infant with a difficult airway.

**Patient concerns:**

A boy with trisomy 18 underwent esophageal banding and gastrostomy for Gross Type C congenital esophageal atresia on postnatal day 3. Tracheostomy was performed on day 49, and definitive surgery for TEF was scheduled on day 83.

**Diagnoses:**

The boy was diagnosed with Gross Type C congenital esophageal atresia shortly after birth.

**Interventions:**

The patient was re-intubated orotracheally in the NICU. Oral insertion of a 4Fr Fogarty catheter to block the TEF was difficult. Insertion through the available tracheostoma was easier.

**Outcomes:**

We confirmed that the catheter could be guided to the TEF entrance using a bronchoscope, and the balloon was expanded to block the TEF. During surgery, there were no problems with airway management.

**Lessons:**

In the management of TEF in children after tracheostomy, if oral insertion of the balloon catheter proves difficult, insertion via the tracheostoma may be effective.

## 1. Introduction

Gross Type C congenital esophageal atresia is associated with tracheoesophageal fistula (TEF). Perioperative airway management, including the prevention of aspiration of gastric contents, is important.^[1]^ Congenital esophageal atresia is generally treated surgically in the early neonatal period, and does not require tracheostomy. In this report, we describe a complicated case with an existing tracheostoma, which could be used to access and block the TEF with a balloon catheter, enabling safe airway management. Written, informed consent was obtained from the patient’s family to publish this case report.

## 2. Case presentation

**Figure 1.**
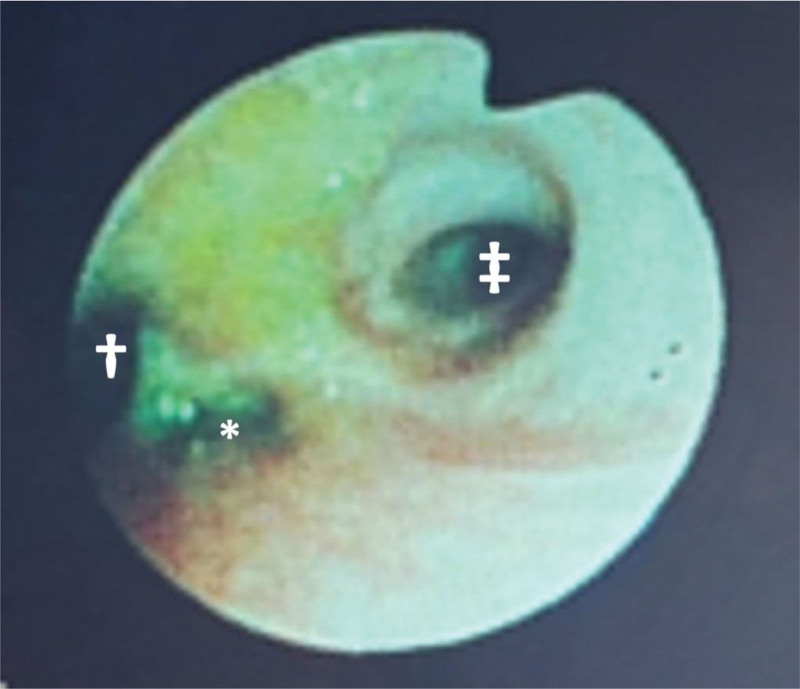
Preoperative bronchoscopy. The TEF was located at the tracheal bifurcation. * TEF; † Left main bronchus; ‡ Right main bronchus.

A male infant was diagnosed with trisomy 18 and Gross Type C esophageal atresia at birth, and was intubated and placed on mechanical ventilation. Extubation was attempted on day 13, but pharyngeal stenosis required reintubation. Tracheostomy was performed on day 49. For esophageal atresia, esophageal banding and gastrostomy were performed on day 3, and definitive esophageal repair with debanding was scheduled on day 83 after birth. The infant had comorbidities including hypertrophic pyloric stenosis (surgically treated on day 55) and muscular ventricular septal defect (VSD), but preoperative echocardiography confirmed that the VSD had almost closed. Because the birth weight was only 1.7 kg and the patient required inotropes and vasopressors for hemodynamic instability until day 7, primary definitive repair was not performed. At the time of preoperative evaluation, the infant was 47cm tall and weighed 2.3 kg. Vital signs, blood tests, electrocardiography, and chest X-ray, showed no significant abnormalities. Preoperative bronchoscopy revealed that the TEF was located at the tracheal bifurcation (Fig. 1). Therefore, airway management was planned using a balloon catheter to block the TEF and prevent aspiration of gastric contents.

### 2.1. Airway management

**Figure 2.**
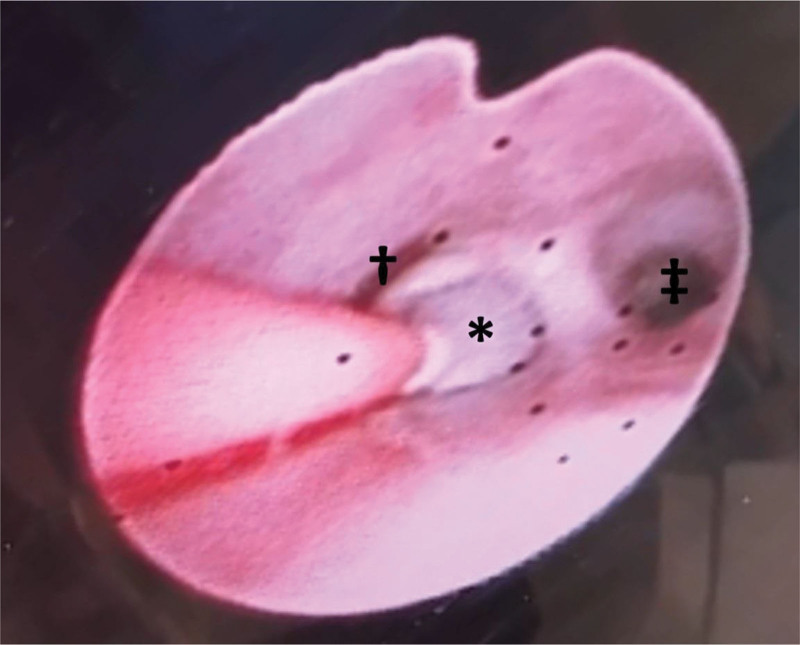
Intraoperative bronchoscopy. The TEF was blocked with a balloon catheter. * TEF; † Left main bronchus; ‡ Right main bronchus.

Before surgery, the tracheostomy tube was replaced with a 3.0mm internal diameter oral endotracheal tube (Microcuff® Avanos Medical Japan Inc., Tokyo, Japan) in the neonatal intensive care unit (NICU). Anesthesia was induced with fentanyl (Fentanyl injection® Terumo Corporation, Tokyo, Japan) 10 µg and rocuronium (Rocuronium bromide® Maruishi Pharmaceutical Co., Ltd., Osaka, Japan) 2.5 mg, followed by multiple attempts to insert a 4Fr Fogarty catheter (Fogarty catheter® Edwards Lifesciences LLC, Irvine, CA, USA) via an oral approach. However, due to the difficulty of laryngeal exposure classified as Cormack grade III, it was challenging to insert the Fogarty catheter through the narrow space between the vocal cords and the endotracheal tube. When insertion was attempted through the covered tracheostoma, the Fogarty catheter was easily advanced. We confirmed that the catheter had been guided to the entrance of the TEF by bronchoscopy, and the balloon was inflated with 0.6 mL of air to block the TEF (Fig. 2). Anesthesia was maintained with sevoflurane (1–2%) and fentanyl, and the surgery proceeded without airway or respiratory complications. After transection of the TEF, the Fogarty catheter was removed, and an end-to-end esophageal anastomosis was performed, completing the surgery. The patient was then transferred back to the NICU. The postoperative course was uneventful, and the patient was discharged on postoperative day 93.

## 3. Discussion

**Figure 3.**
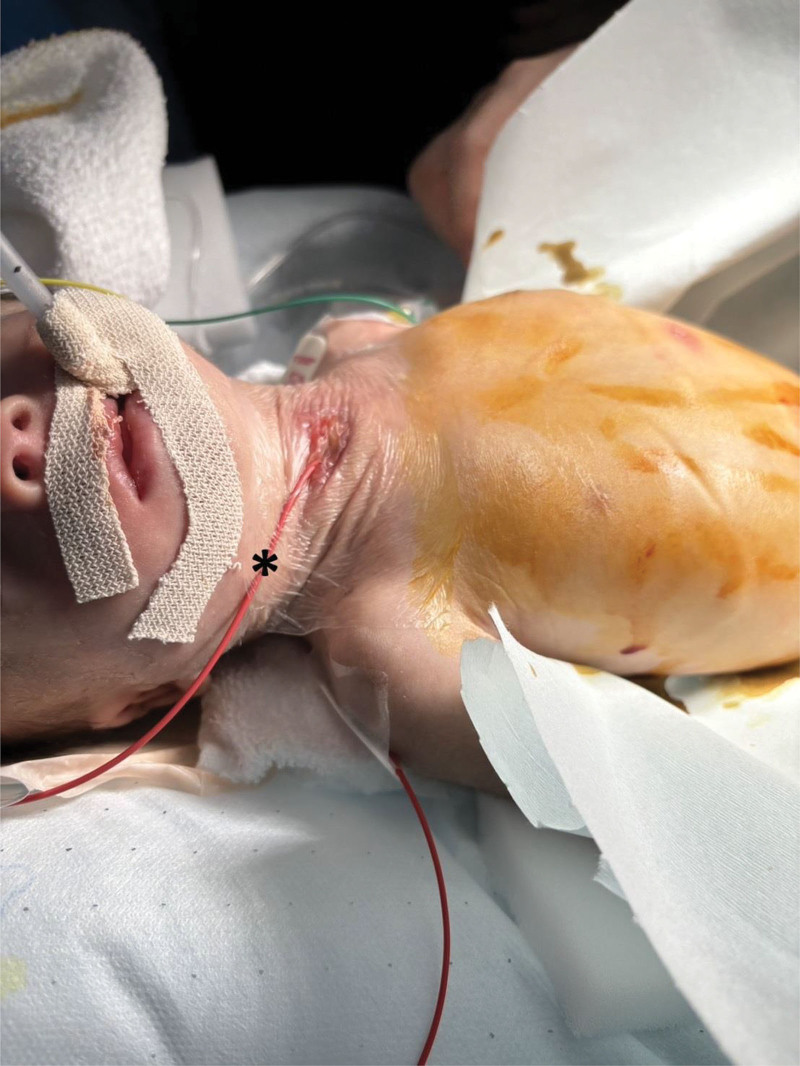
Securement of balloon catheter. The balloon catheter (*) was secured with adhesive transparent film dressing.

There are 2 major methods for managing TEF during definitive repair of esophageal atresia. One approach involves blocking the TEF using a balloon catheter, such as a bronchial blocker or a Fogarty catheter. The other approach involves placing the tip of an endotracheal tube distal to the TEF, such as by bronchial intubation.^[1,2]^ In the reported case, the surgeon requested occlusion by balloon catheter to facilitate identification of the TEF, and airway management was performed using the former approach. Insertion of a balloon catheter for this purpose is typically performed via an oral approach using laryngoscopy. However, in this case, the insertion was difficult. Insertion through the tracheostoma allowed easy placement into the trachea and successful TEF occlusion without any issues. The catheter was secured with adhesive transparent film dressing, which allowed for stable intraoperative management without significant displacement (Fig. 3). An important consideration when using a Fogarty catheter is the balloon volume. Unlike bronchial blockers, the Fogarty catheter has a low-volume, high-pressure balloon, so it is essential to use a bronchoscope to monitor the catheter’s position and inflation to avoid airway mucosal injury and the balloon rupture.^[3]^ Patients undergoing TEF repair typically do not have a tracheostoma. Our experience inserting a balloon catheter via the tracheostoma was technically straightforward. However, the pediatric tracheostoma is fragile and prone to granuloma formation. It has been reported that complications such as subcutaneous misplacement, tracheal laceration, and tube obstruction due to granulomas can occur in 3–19% of cases, requiring careful attention.^[4,5]^ While not intended for TEF management, guidelines exist for 1-lung ventilation in pediatric patients with a tracheostoma. Templeton et al recommended inserting a bronchial blocker through the tracheostoma and placing an endotracheal tube adjacent to it.^[6]^ Similarly, there are reports of successful 1-lung ventilation in adult patients with tracheostoma stenosis, achieved by placing a thin endotracheal tube alongside a bronchial blocker.^[7]^ Demirkol et al also reported establishing lung isolation by inserting 2 endotracheal tubes through a tracheostoma.^[8]^

In the reported case, the tracheostomy tube was replaced with an oral endotracheal tube in the NICU prior to transfer to the operating room. Given that children with trisomy 18 often have a difficult airway, the risk associated with replacing the tracheostomy tube with an oral endotracheal tube was considerable.^[9]^ However, intraoperative airway management using a more conventional and familiar approach was considered preferable, and an oral endotracheal tube was thought to provide more secure access for bronchoscopy and suctioning during surgery compared to a tracheostomy tube. It is worth considering whether TEF management could have been achieved by inserting the Fogarty catheter alongside the existing tracheostomy tube without replacing it with an oral endotracheal tube. It is possible that inserting an endotracheal tube through the tracheostoma might have been a safer approach.

Based on the above, although this method is limited to patients with an existing tracheostoma, it may serve as an effective strategy for TEF management in children with anticipated difficult airways, such as those with trisomy 18.

## 4. Conclusion

When managing TEF in pediatric patients with a tracheostoma, inserting a balloon catheter through the tracheostoma should be considered as a useful option. Although this approach requires caution due to potential complications, it is technically straightforward.

## Author contributions

**Data curation:** Masato Ryo, Maiko Hosokawa, Kiyoko Bito.

**Project administration:** Masato Ryo.

**Writing – original draft:** Masato Ryo.

**Writing – review & editing:** Masato Ryo, Yusuke Ishida, Maiko Hosokawa, Kiyoko Bito.

**Supervision:** Yusuke Ishida, Maiko Hosokawa, Kiyoko Bito, Katsunori Oe.
